# Microbiota diversity and stability of the preterm neonatal ileum and colon of two infants

**DOI:** 10.1002/mbo3.64

**Published:** 2013-01-24

**Authors:** Eoin Barrett, Caitriona M Guinane, C Anthony Ryan, Eugene M Dempsey, Brendan P Murphy, Paul W O'Toole, Gerald F Fitzgerald, Paul D Cotter, R Paul Ross, Catherine Stanton

**Affiliations:** 1 Food Biosciences, Teagasc Food Research Centre, Moorepark, Fermoy Co.Cork, Ireland; 2 Alimentary Pharmabiotic Centre, University College CorkCork, Ireland; 3 Department of Paediatric and Child Health, University College Cork, National University of IrelandCork, Ireland; 4 Department of Microbiology, University College Cork, National University of IrelandCork, Ireland

**Keywords:** Colostomy, gastrointestinal tract, ileostomy, microbiota, preterm infant

## Abstract

The composition of the microbiota associated with the human ileum and colon in the early weeks of life of two preterm infants was examined, with particular emphasis on the *Lactobacillus* and *Bifidobacterium* members. Culturing work showed that bifidobacteria and lactobacilli in the ileostomy changed over time, compared with the colostomy effluent where there was far less variation. The colostomy infant was dominated by two phyla, Actinobacteria and Firmicutes, while in the ileostomy samples, Proteobacteria emerged at the expense of Actinobacteria. Bacteroidetes were only detected following the reversal of the ileostomy in the final fecal sample and were not detected in any colonic fluid samples. Clostridia levels were unstable in the colostomy fluid, suggesting that the ileostomy/colostomy itself influenced the gut microbiota, in particular the strict anaerobes. Pyrosequencing analysis of microbiota composition indicated that bifidobacteria and lactobacilli are among the dominant genera in both the ileal and colonic fluids. Bifidobacteria and lactobacilli levels were unstable in the ileostomy fluid, with large reductions in numbers and relative proportions of both observed. These decreases were characterized by an increase in proportions of *Streptococcus* and Enterobacteriaceae. *Clostridium* was detected only in the colonic effluent, with large changes in the relative proportions over time.

## Introduction

The gastrointestinal tract (GIT) in newborn infants is almost sterile. However, immediately after birth, microbes acquired from the mother and the surrounding environment colonize the gut of the term infant, with a dense and intricate microbiota subsequently developing (Rotimi and Duerden 1981; DiGiulio et al. 2008; Jimenez et al. 2008; Marques et al. 2010; O'Toole and Claesson 2010). Indeed, colonization and microbial composition of the neonatal gut are influenced by a number of factors, including mode of delivery, dietary regime, antibiotic use, and environmental factors (Biasucci et al. 2008; Adlerberth and Wold 2009). In full-term vaginally delivered infants, microorganisms such as enterobacteria, staphylococci, and streptococci are the earliest to colonize (Stark and Lee 1982; Mackie et al. 1999; Penders et al. 2006; Morelli 2008), followed by the strict anaerobes, *Clostridium*, *Bacteroides*, and bifidobacteria, with bifidobacteria becoming dominant in the infant gut (Harmsen et al. 2000; Penders et al. 2006). At 1 month of age, bifidobacteria were detected in almost all infants, whereas the prevalence of lactobacilli in infants was ∼30% (Penders et al. 2006).

An infant is considered preterm if born before the 37th week of gestation (Dennison 1976). The intestinal microbiota of preterm infants differs from that of term infants, with a number of factors, including immature gut motility, delayed nutrition, and antibiotic use influencing colonization and often delaying colonization by bifidobacteria (Stark and Lee 1982; Gewolb et al. 1999; Hoy et al. 2000; Fanaro et al. 2003). In addition, it has been reported that the pH of the stomach, an important physiochemical barrier (Martinsen et al. 2005; Dial 2009) of preterm infants is higher than that of term infants (Washington et al. 1999; Mitchell et al. 2001). It has been suggested that the higher gastric pH of preterm infants is due to more frequent feeding than full-term infants and an inferior gastric acid secretion mechanism possibly leading to a greater risk of bacterial infections (Sondheimer and Clark 1985; Sondheimer et al. 1985; Sutphen and Dillard 1986; Newell et al. 1988). Necrotizing enterocolitis (NEC) is a common cause of morbidity and mortality in infants, particularly in preterm infants (Deshpande et al. 2007; Neu and Walker 2011). It is a life-threatening illness of the GIT which causes inflammation and necrosis of the intestine and can affect other organs of the body, including the brain. NEC affects 5–10% of infants born with a birth weight of 500–1500 g (Erasmus et al. 2002; Horbar et al. 2002; Holman et al. 2006) and the condition is fatal for 20–30% of cases, with the highest rate of mortality among those infants requiring surgery (Fitzgibbons et al. 2009).

The human microbiota has a significant influence on human health and has been implicated in a number of disorders including obesity and inflammatory bowel diseases (Ley et al. 2006; Peterson et al. 2008). Bifidobacteria and lactobacilli are considered among the most important health-promoting bacteria in humans as well as being the best characterized probiotics (Rastall 2004; O'Flaherty and Klaenhammer 2010). Studies have shown that they can contribute to digestion, immune stimulation, and inhibition of pathogens in addition to producing a large number of metabolites that may be beneficial to the human host (Deguchi et al. 1985; Fuller 1989; Cerning 1995; Sreekumar and Hosono 1998; Hou et al. 2000; Ogawa et al. 2001; Crittenden et al. 2003; Coakley et al. 2006). Bifidobacteria are of great importance in early life and can become the dominant genus in the gut, outnumbering all other bacterial groups and species (Chierici et al. 2003; Penders et al. 2006), with *Bifidobacterium longum* and *Bifidobacterium breve* the most commonly reported (Satokari et al. 2002; Haarman and Knol 2005; Mikami et al. 2009). However, low numbers of bifidobacteria have been reported in the preterm infant gut, with gestational age identified as a major determinant of bifidobacterial colonization (Westerbeek et al. 2006; Butel et al. 2007). The prevalence and numbers of lactobacilli were not as high as those reported for bifidobacteria in stools of 1-month-old term infants, with significantly higher numbers of lactobacilli in stools of exclusively formula-fed infants compared with exclusively breast-fed infants (Penders et al. 2006). *Lactobacillus rhamnosus* and *Lactobacillus gasseri* are the most commonly reported species (Ahrne et al. 2005) and similar to trends for bifidobacteria, low numbers of lactobacilli have been reported for preterm infants (Westerbeek et al. 2006; Chang et al. 2011; Arboleya et al. 2012).

The different sections of the GIT harbor unique microbial populations (Mattila-Sandholm et al. 2002), and while extensive research has been conducted on the oral cavity and colon, little is known about the enclosed ecosystems of the GIT, with most of the current knowledge derived from tissues that were taken during colonoscopy, samples collected following small bowel transplantations, or from sudden death victims (Booijink et al. 2010). The aim of this study was to observe the microbial diversity of the ileum and colon of two infants, with particular focus on lactobacilli and bifidobacteria.

## Experimental Procedures

Ethical Approval for the study was received from the Clinical Research Ethics Committee of the Cork Teaching Hospitals, Cork, and fully informed consent was obtained from all parents prior to initiation of the study.

### Patients

“Infant 1” was a preterm male, born spontaneously following 26 weeks' gestation by vaginal delivery, weighing 1040 g. The infant was treated for sepsis at 3 days of age and received antibiotic treatment for the first 13 days of life (fluconazole, teichoplanin, gentamicin, and benzylpenicillin) in addition to receiving antibiotics on days 36–39 (fluconazole, teichoplanin, cefotaxamine, gentamicin, and benzylpenicillin). Infant 1 was treated for suspected NEC at day 7 of life and following an intestinal perforation underwent an exploratory laparotomy and the creation of a stoma at day 13 of life. The ileostomy was reversed and intestinal continuity restored at 79 days of age. Six ileostomy samples were obtained from the infant; samples were taken on days 43, 50, 57, 64, 71, and 78. Three fecal samples were collected from the infant at 105, 117, and 217 days of age. Infant 1 was fed with expressed breast milk.

“Infant 2” was a preterm male born following 32 weeks' gestation by cesarean section, weighing 1200 g. At day 3 of life, the infant suffered an intestinal perforation. This required a laparotomy and the creation of a stoma. Infant 2 received antibiotic treatment (benzylpenicillin, gentamicin, and metronidazole) on days 3–13 of life and a different antibiotic treatment (fucidin) on days 27–32. Nine colostomy samples were obtained (DNA was extracted from 8); samples were taken on days 34, 42, 48, 70, 77, 96, 119, 152, and 210 of age. The infant had received three vaccinations over the period of the study. On day 68, the infant received the six-in-one vaccine (*Corynebacterium diphtheriae* toxin, *Clostridium tetani* toxin, *Bordetella pertussis* toxin, *Poliovirus* (inactivated), *Hepatitis B* antigens, and *Haemophilus influenzae* polysaccharide) and PCV (pneumococcal conjugate vaccine, containing a pneumococcal polysaccharide) vaccine, while on day 96 the infant received the BCG (Bacillus Calmette-Guérin, viable attenuated *Mycobacterium bovis*) vaccination followed by the men C (containing nonviable *Neisseria meningitidis*) and six-in-one vaccination on day 138 of life. Infant 2 was breast-fed and began consuming infant formula in addition to breast-feeding on day 165 of life and had started receiving solid food on day 195 of life. We have no information regarding whether the mothers or infants received probiotics in their diet.

### Ileal/colon fluid sampling

In all cases, putative bifidobacteria and lactobacilli were enumerated from the ileal/colon fluid samples. Ileal/colon fluid samples were stored at 5°C and were processed in the laboratory within 5 h of sampling. Ileal/colon fluid samples were mixed by vortexing in maximum recovery diluent (Oxoid, Ltd., Hampshire, UK), serially diluted and spread plated on the appropriate medium and incubated as described below. Bifidobacteria and lactobacilli were enumerated based on colony morphology and confirmed by microscopy.

### Media and growth conditions

Serial dilutions of fecal samples were spread plated onto TOS-propionate agar (Yakult Pharmaceutical Industry Co., Ltd., Toyko, Japan) supplemented with 100 μg/mL of Mupirocin (Oxoid), added as antimicrobial susceptibility disks to molten agar as previously described (Simpson et al. 2003), to preselect for bifidobacteria and *Lactobacillus* selective agar (LBS; Becton Dickinson Co., Cockeysville, MD) to preselect for lactobacilli. Agar plates were incubated anaerobically (anaerobic jars with Anaerocult®A gas packs; Merck, Darmstadt, Germany) at 37°C for 5 days.

### Generation of 16S rRNA gene amplicons for high-throughput sequencing

The generation of 16S rRNA gene amplicons was performed as described previously (O'Sullivan et al. 2011). DNA was extracted from samples according to a previously described protocol (Zoetendal et al. 2006) using the QIAamp DNA Stool Mini kit (Qiagen, West Sussex, UK). Universal 16S rRNA gene primers, as previously described (Murphy et al. 2010), were used to amplify from highly conserved regions corresponding to those flanking the V4 region. Pyrosequencing was performed at the Teagasc 454 Sequencing facility on a Genome Sequencer FLX platform (Roche Diagnostics Ltd., West Sussex, UK) according to the manufacturer's protocols.

### Bioinformatic analysis

Raw 16S rRNA gene sequencing reads were initially quality trimmed using a locally installed version of the Ribosomal Database Project (RDP) Pyrosequencing Pipeline applying the criteria as previously described (O'Sullivan et al. 2011). Trimmed FASTA sequences were subsequently BLASTed (Altschul et al. 1997) against a locally installed version of SILVA 16S rRNA database (http://www.arb-silva.de). The output from the BLAST analysis was then parsed using MEGAN software (version 4.6) (Huson et al. 2007) using modified accession look-up tables for mapping the SILVA assignments to NCBI taxonomy. Briefly, bit scores from within MEGAN were used to filter the results prior to tree construction and summarization. A bit-score of 86 was selected, as previously used for 16S rRNA gene sequence data (Urich et al. 2008). Phylum, family, and genus counts for each subject were extracted from MEGAN. The MOTHUR software package was then used for clustering and diversity analysis of the sequence data (Schloss and Handelsman 2008).

## Results

In this study, the microbial diversity of ileal and colonic fluid of two preterm infants was reported, with particular emphasis on the *Lactobacillus* and *Bifidobacterium* populations. The infant ileostomy/colostomy offers a rare opportunity to explore the microbiota in the interluminal fluid of the newborn. Samples were collected over time, and in the case of one infant, fecal samples were taken following ileostomy reversal. This allowed us to examine the microbial colonization of the small intestine and colon at an early stage of life.

### Enumeration of bifidobacteria and lactobacilli by culture-dependent methods overtime

Initially, culturing techniques were used to enumerate the bifidobacteria and lactobacilli populations in both the ileal and colon effluents by plating the samples on *Bifidobacterium* and *Lactobacillus* selective media.

#### Infant 1

**Figure 1:**
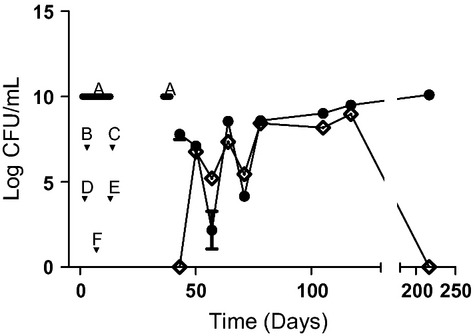
Enumeration of *Lactobacillus* and *Bifidobacterium* in ileal fluid over time. • Log *Bifidobacterium* cfu/mL; ◊ Log *Lactobacillus* cfu/mL. A – administration of antibiotics, B – sepsis, suspected necrotizing enterocolitis and administration of antibiotics, C – laparotomy, D – donor milk, E – intestinal perforation, and F – necrotizing enterocolitis.

The bifidobacteria and lactobacilli numbers changed over time (Fig. 1). On days 36–39, the infant had received antibiotic treatment (fluconazole, teichoplanin, cefotaxamine, gentamicin, and benzylpenicillin), and subsequently, lactobacilli were not detected in the ileal fluid at day 43, whereas bifidobacteria were detected at 9.7 × 10^7^ cfu/mL. Culturable lactobacilli were detected on day 50 (7.0 × 10^6^ cfu/mL), and subsequently, there was a reduction in *Bifidobacterium* and *Lactobacillus* numbers in the ileal fluid on day 57 (1.6 × 10^3^ and 1.6 × 10^5^ cfu/mL, respectively). On days 57 and 64, samples were very viscous when compared with all other ileal fluid samples. There was an increase in both *Lactobacillus* and *Bifidobacterium* numbers from day 57 (to 4.1 × 10^8^ and 2.7 × 10^7^ cfu/mL, respectively). On day 71, there was a reduction in *Bifidobacterium* and *Lactobacillus* numbers to 1.7 × 10^4^ and 2.9 × 10^5^ cfu/mL, respectively, followed by an increase on day 78 to 3.9 × 10^8^ and 3.0 × 10^8^ cfu/mL, respectively. Numbers of lactobacilli and bifidobacteria were similar in the fecal samples collected on days 105 and 117, following reversal of the ileostomy on day 79; however, on day 217, lactobacilli were undetectable while bifidobacteria reached their highest numbers (1.3 × 10^10^ cfu/g) over the duration of the study.

#### Infant 2

**Figure 2:**
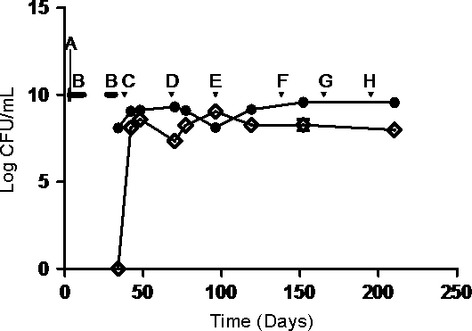
Enumeration of *Lactobacillus* and *Bifidobacterium* in colon fluid over time. • Log *Bifidobacterium* cfu/mL; ◊ Log *Lactobacillus* cfu/mL. A – laporotomy, B – administration of antibiotics, C – breast milk fortifier, D – six-in-one and PVC vaccination, E – vaccination milk, F – six-in-one vaccination, G – infant formula, and H – solid food.

There was less variation in bifidobacterial and lactobacilli numbers over time (Fig. 2). On days 27–32, the infant received antibiotic treatment (fucidin) and subsequently on day 34, bifidobacteria were detected at 1.6 × 10^8^ cfu/mL in the colonic fluid, while lactobacilli were not detected. Bifidobacterial numbers increased on day 42 to 1.1 × 10^9^ cfu/mL and indeed, bifidobacterial numbers remained >10^9^ cfu/mL for all but one of the remaining samples. On day 96, the bifidobacterial numbers decreased to 1.3 × 10^8^ cfu/mL but increased to 1.5 × 10^9^ cfu/mL by day 119. Lactobacilli were detected at 1.3 × 10^8^ cfu/mL on day 42, reduced to a minimum of 2.3 × 10^7^ cfu/mL on day 70, and increased to a maximum of 1.2 × 10^9^ cfu/mL on day 96.

### Composition of the gut microbiota of ileostomy and colostomy patients over time

The microbial composition of the ileal/colon fluid of the two infants was elucidated through high-throughput pyrosequencing (Roche-454 Titanium) of 16S rRNA (V4) gene amplicons.

#### Infant 1

**Figure 3:**
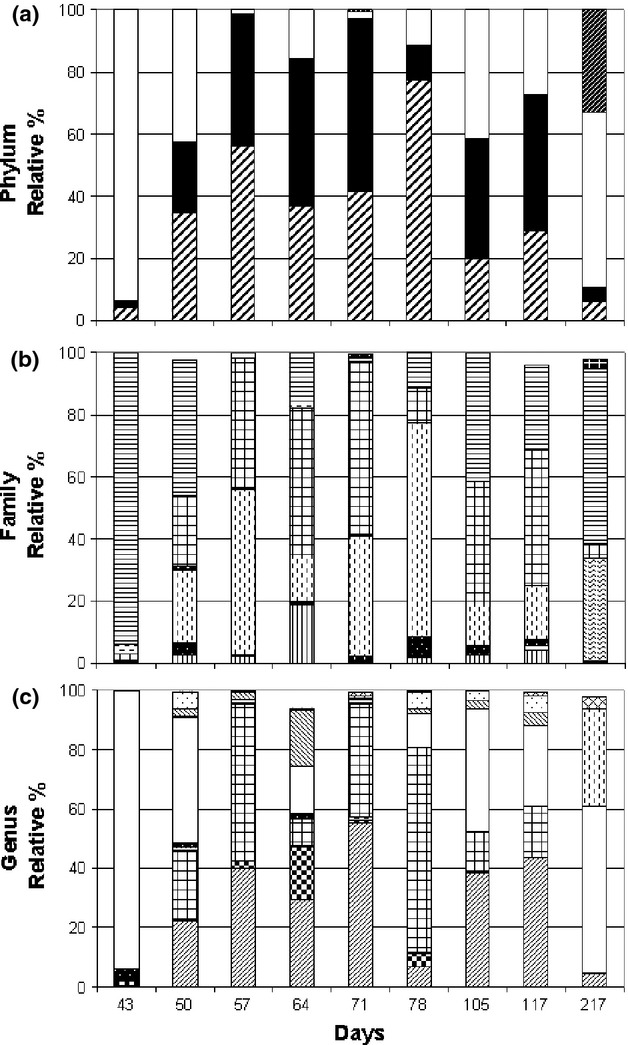
The relative proportion of bacteria isolated from ileal fluid over time as determined by 16s rRNA gene amplicon sequencing. (a) Relative proportion of phyla over time, Actinobacteria 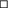; Proteobacteria 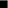; Firmicutes 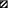; *Bacteroidetes* 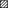. (b) Relative proportion of families over time, Enterobacteriaceae 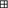; Veillonellaceae 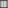; Clostridiaceae 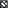; Streptococcaceae 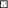; Bifidobacteriaceae 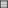; Lactobacillaceae 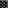; other families 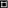; Enterococcaceae 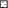; Bacteroidaceae 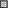; Staphyloccaceae 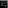. (c) Relative proportion of genera over time, *Bifidobacterium* 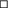; Enterobacteriaceae-associated genera 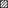; *Haemophilus* 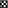; *Bacteroides* 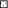; *Streptococcus* 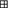; *Veillonella* 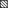; *Lactobacillus* 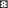; other genera 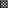; *Enterococcus* 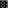; *Leuconostoc* 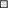; *Staphylococcus* 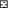.

Taxonomy-based analysis showed that at the phylum level the ileum gut microbiota changed over the time course of this study (Fig. 3a). Initially, at day 43, the ileal fluid was dominated by Actinobacteria (94%), followed by Firmicutes (4%) and Proteobacteria (2%) (Fig. 3a). At the family level, the dominant groups were Bifidobacteriaceae (94%), followed by Enterococcaceae (3%) and Enterobacteriaceae (2%) (Fig. 3b). The Bifidobacteriaceae consisted exclusively of bacteria from the genus *Bifidobacterium* (Fig. 3). Other dominant groups at the genus level were *Enterococcus* (3%) and *Haemophilus* (1%). At day 50, there was a decline, at phylum level, in the dominance of the Actinobacteria (43%), with the same percentage reduction seen in Bifidobacteriaceae and *Bifidobacterium* proportions at family and genus levels, respectively. This coincided with increases in abundance at a family level of Streptococcaceae (24%) and Enterobacteriaceae (23%) (Fig. 3b). These increases were noted at genus level, with increases in the relative proportions of *Streptococcus* (23%) and Enterobacteriaceae-associated genera (22%) (Fig. 3b and c). *Lactobacillus* also increased to 5% of the relative proportion of the assignable reads, while *Veillonella* increased to 2% and *Enterococcus* decreased to 1%. The decline observed in culturable bifidobacterial numbers at day 57 was mirrored by a decline in the relative proportion of Actinobacteria (from 94% on day 43 to 1.5% at day 57), with Firmicutes and Proteobacteria increasing to 56% and 42% (Fig. 3a), respectively. At the genus level, there was a decline in the relative proportion of *Bifidobacterium* to 1.4%, while the relative proportions of *Streptococcus* (53%), Enterobacteriaceae-associated genera (42%), *Veillonella* (2%), and *Haemophilus* (2%) continued to increase (Fig. 3b and c). On day 64, there was an increase in culturable numbers of both lactobacilli and bifidobacteria; however, the relative proportions of Firmicutes and Proteobacteria remained dominant at 37% and 47% (Fig. 3a), respectively. Furthermore, at a genus level, the relative proportions of lactobacilli and bifidobacteria reached 0.5% and 16% of the assignable reads, respectively. The relative proportion of *Streptococcus* (9%) decreased, while Enterobacteriaceae-associated genera (47%), *Veillonella* (19%), and *Enterococcus* (2%) increased (Fig. 3b and c). The proportion of Actinobacteria decreased from 16% on day 64 to 2% on day 71, with Firmicutes and Proteobacteria increasing to 41% and 56% (Fig. 3a), respectively. At family and genus levels, the dominant groups were Enterobacteriaceae (56%) and *Streptococcus* (38%), followed by *Bifidobacterium* (1.5%). On day 78, the ileal fluid was dominated by Firmicutes (77%) followed by Proteobacteria (11%) and Actinobacteria (11%) (Fig. 3a), while at family-level Enterobacteriaceae levels decreased (11%). At genus level, the relative proportions of *Streptococcus* (69%), *Bifidobacterium* (11%), *Lactobacillus* (6%), and *Haemophilus* (4%) increased and *Veillonella* (2%) decreased. Following reversal of the ileostomy on day 79, the relative proportions of Actinobacteria, Proteobacteria, and Firmicutes in the fecal sample collected on days 105 reached 42%, 39%, and 20%, respectively, while at a family-level Bifidobacteriaceae (42%), Enterobacteriaceae (39%), Streptococcaceae (14%), Lactobacillaceae (3%), and Veillonellaceae (3%) dominated. At day 117, the fecal sample was dominated by Proteobacteria (44%), followed by Firmicutes (29%) and Actinobacteria (27%) (Fig. 3a). At the family level, the dominant groups were Enterobacteriaceae (44%), Bifidobacteriaceae (27%), followed by Streptococcaceae (17%) (Fig. 3b). The Bifidobacteriaceae consisted exclusively of bacteria from the genus *Bifidobacterium* (Fig. 3). Other dominant groups at the genus level were *Streptococcus* (17%), *Lactobacillus* (5%), *Veillonella* (4%), and *Clostridium* (1%). At phylum level, the final fecal sample was dominated by Actinobacteria and Bacteroidetes, reflected by the relative dominance of *Bifidobacterium* and *Bacteroides* at genus level (56% and 33%, respectively) (Fig. 3a and c). Interestingly, *Bacteroides* were detected in the final fecal sample analyzed, which was the first time this was found in either ileal or fecal samples from the infant. The disappearance of *Lactobacillus* in the final fecal sample mirrors the result seen in the culturing work (Fig. 3).

#### Infant 2

**Figure 4:**
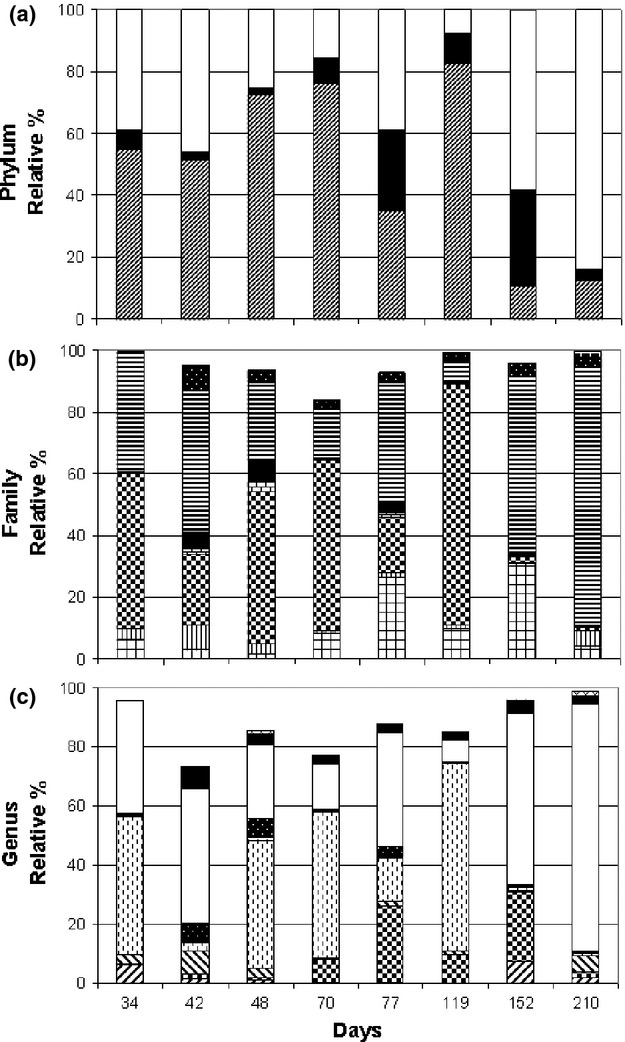
The relative proportion of bacteria isolated from colon fluid over time as determined by 16s rRNA gene amplicon sequencing. (a) The relative proportion of phyla over time, Actinobacteria 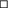; Proteobacteria 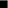; Firmicutes 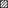. (b) Relative proportion of families over time, Enterobacteriaceae 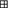; Veillonellaceae 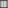; Clostridiaceae 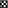; Clostridiales Family XI incertae sedis 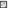; Streptococcaceae 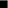; Bifidobacteriaceae 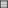; Lactobacillaceae 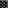; other families 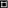. (c) Proportion of genera over time, *Bifidobacterium* 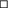; Enterobacteriaceae-associated genera 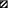; *Haemophilus* 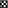; Streptococcus 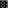; *Veillonella* 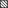 *Lactobacillus* 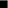; other genera 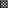; *Enterococcus* 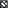; *Anaerococcus* 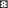; *Clostridium* 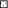.

Pyrosequencing revealed that at the phylum level, the colonic microbiota changed over the time course of this study (Fig. 4). On days 27–32, the infant had received antibiotic treatment (fucidin), and subsequently at day 34, bifidobacteria were detected at the lowest numbers in the colonic fluid throughout this study. At day 34, the colonic fluid was dominated by Firmicutes (54%), followed by Actinobacteria (39%) and Proteobacteria (7%), with no Bacteroidetes detected (Fig. 4a). Indeed, it is a feature of the “colostomy” infant that Bacteroidetes were not detected in any sample analyzed throughout the study. At the family level, the most dominant groups of the assignable reads were Clostridiaceae (50%), followed by Bifidobacteriaceae (39%) and Enterobacteriaceae (7%), with *Clostridium* and *Bifidobacterium* dominating at genus level (47% and 38%, respectively) followed by *Veillonella* (3%) (Fig. 4b and c). At day 42, there were slight decreases at phylum level, in Firmicutes (51%) and Proteobacteria (3%), while the Actinobacteria increased (46%). This coincided with an increase at family level of Bifidobacteriaceae (45%) and a decrease in Clostridiaceae (23%). These changes were seen at genus level with the relative proportion of *Bifidobacterium* increasing to 45%, the species *Clostridium* decreasing from 47% to 3%, and the emergence of *Lactobacillus* at 7% (Fig. 4a–c). At this sampling stage, while over 95% of the sequences were assigned at family level, 26% of the sequences could not be assigned to a genus. By day 48, the proportion of Actinobacteria decreased from 47% on day 42 to 25%, with Firmicutes increasing to 73% (Fig. 4a). At family and genus levels, the dominant groups were Clostridiaceae (49%), Bifidobacteriaceae (25%), Streptococcaceae (7%), Lactobacillaceae (4%), Clostridiales Family XI incertae sedis (3%), and Veillonellaceae (3%); and *Clostridium* (43%), *Bifidobacterium* (25%), *Streptococcus* (6%), *Lactobacillus* (4%), and *Veillonella* (3%), respectively. The Firmicutes were again dominant in the colonic sample, reaching over 76%, with the Actinobacteria proportion decreasing to 15% and Proteobacteria increasing to 8% on day 70 (Fig. 4a). At family level, Clostridiaceae and Enterobacteriaceae increased (56% and 8%, respectively), while Bifidobacteriaceae (15%), Streptococcaceae (0.5%), Lactobacillaceae (3%), Clostridiales Family XI incertae sedis (0.3%), and Veillonellaceae (0.6%) decreased from day 48. At genus level, the relative proportions of *Streptococcus* (0.5%), *Bifidobacterium* (15%), *Lactobacillus* (3%), and *Veillonella* (0.6%) decreased, while *Clostridium* (49%) and *Haemophilus*, increased. On day 77, the relative proportions of Actinobacteria, Proteobacteria, and Firmicutes in the colonic fluid sample reached 39%, 26%, and 35%, respectively, while at family-level Bifidobacteriaceae (39%), Enterobacteriaceae (26%), Clostridiaceae (18%), Streptococcaceae (4%), Lactobacillaceae (3%), and Veillonellaceae (2%) dominated. The Bifidobacteriaceae consisted exclusively of bacteria from the genus *Bifidobacterium* (Fig. 4). At day 119, the colonic fluid was dominated by Firmicutes (82%), followed by Proteobacteria (10%) and Actinobacteria (8%) (Fig. 4a). At the family level, the most dominant groups of the assignable reads were Clostridiaceae (78%), followed by Enterobacteriaceae (10%) and Bifidobacteriaceae (8%), with *Clostridium, Haemophilus*, and *Bifidobacterium* dominating at genus level (70%, 10%, and 7%, respectively) followed by *Lactobacillus* (2%) (Fig. 4b and c). At day 152, there was a decline at phylum level, in the dominance of the Firmicutes (11%), with reductions seen in Clostridiaceae (2%) and *Clostridium* (1%) proportions at family and genus levels, respectively. This coincided with increases in abundance at a family level of Bifidobacteriaceae (58%) and Enterobacteriaceae (31%) (Fig. 4b). These increases were noted at genus level, in particular with increases in the relative proportions of *Bifidobacterium* (58%) (Fig. 4b and c). *Lactobacillus* also increased to 4% of the relative proportion of the assignable reads. At phylum level, the final colonic sample was dominated by Actinobacteria (84%), with the same percentage increase seen in Bifidobacteriaceae and *Bifidobacterium* proportions at family and genus levels, respectively.

## Discussion

This study gives the first insight into the composition of the microbiota of two preterm infants, as well as highlighting the dynamic changes which occur in the premature newborn human ileum and colon. It also shows that the microbiota of the neonatal ileum/colon in ileostomy/colostomy infants is dynamic and unstable, with large changes observed at genus level over the duration of this study; however, the obvious limitations associated with this study are (1) the number of subjects, (2) the health status of the infants, and (3) the fact that this study is observational should be noted.

The microbiota of breast-fed, full-term, vaginally delivered healthy infants is dominated by bifidobacteria and regarded as ideal for infants, whereas in preterm infants, it is characterized by decreases in *Bifidobacterium* and *Bacteroides* and increases in *Clostridium* and *Staphylococcus* populations (Penders et al. 2006; Chang et al. 2011). At phylum level, the preterm microbiota in the first 4 weeks of life is dominated by Proteobacteria, with a high level of interindividual variation (Mshvildadze et al. 2010; Chang et al. 2011). La Tuga et al. (2011) also reported the dominance of Proteobacteria and Firmicutes in extremely low-birth-weight premature infants in the first month of life, while a link between antibiotic use and the disturbed microbial colonization in preterm infants has been reported (Madan et al. 2012). In this study, we found *Bifidobacterium* and *Lactobacillus* to be among the dominant genera in the preterm colon and ileum of infants with serious health issues, although sampling did not commence until weeks 5 and 6 of life for the colostomy and ileostomy infants, respectively. Koenig et al. (2011), using metagenomic analysis, found a abundance of Actinobacteria genes in a full-term breast-fed infant on days 92–118 of life. Functional genes belonging to Bacteroidetes emerged on the introduction of plant-derived foods. At phylum level, Bacteroidetes were only detected following the reversal of the ileostomy in the final fecal sample from infant 1 and were not detected in any colonic fluid samples. This indicates that the genus *Bacteroides*, a very important genus in adult humans (Wexler 2007) which dominates the neonatal gut along with other strict anaerobes *Bifidobacterium* and *Clostridium* (Penders et al. 2006; Morelli 2008; Adlerberth and Wold 2009), was absent from the ileum and colon of both infants undergoing ileostomy/colostomy.

In the intestine of preterm infants, the microbial colonization pattern is disturbed (Hoy et al. 2000; Fanaro et al. 2003; Westerbeek et al. 2006); however, the significance of this abnormal colonization is unclear (Bjorkstrom et al. 2009). It has been suggested that birth gestational age is a major factor in bifidobacterial colonization in preterm infants (Butel et al. 2007). In this study, both the ileostomy and colostomy infants harbored bifidobacteria by days 43 and 34 of life, respectively. Indeed, a potential mechanism whereby bifidobacteria and lactobacilli may positively influence health is in the treatment or prevention of NEC (Embleton and Yates 2008; Mshvildadze and Neu 2009; Morowitz et al. 2010). While the causative agents of NEC are unknown, pathogenic bacteria are suspected to play a role. However, until recently, no specific pathogen had been identified as causative for NEC, as some of the study outcomes are conflicting, suggesting that no single causative bacterium is responsible (Wang et al. 2009; Morowitz et al. 2010). Proteobacteria have previously been implicated in the development of NEC (Wang et al. 2009; Mai et al. 2011). We identified an increase in *Streptococcus* and Enterobacteriaceae proportions in the infant, coinciding with a decrease in *Bifidobacterium* and *Lactobacillus* numbers and relative proportions; however, the infant did not develop NEC. It has been suggested that there may be a link between low *Bifidobacterium* and *Lactobacillus* numbers in preterm infants and the development of NEC (Caplan 2009). However, Morowitz et al. (2010) investigated why more than 90% of preterm infants lack those desirable bugs but do not develop the disease. In the case of the “ileostomy infant” in this study, bifidobacteria were initially dominant in the ileum (>90%). However, by day 71, they constituted <1.5% of total bacteria, with *Streptococcus* and Enterobacteriaceae dominating. This would suggest that a high level of Proteobacteria and the absence of *Bifidobacterium* and *Lactobacillus* alone are not sufficient to cause NEC.

Previous work has reported high levels of the facultative anaerobes lactobacilli and enterobacteria in the ileostomy effluent of adults rather than the expected strict anaerobes, *Clostridium* and *Bacteroides*, while the greatest influence on the ileum microbiota was the ileostomy itself allowing oxygen into the environment (Hartman et al. 2009). In this study, we noted a similar occurrence with the absence of the strict anaerobe *Bacteroides* in all colostomy samples and all but the final fecal sample from the ileostomy infant, which was collected 132 days after reversal of the ileostomy. However, high numbers (>10^9^) of the more aero-tolerant bifidobacteria were detected. Hartman et al. (2009) did not report the presence of bifidobacteria in the ileal fluid of the adults also suffering from serious illnesses and injuries of the GIT. The relative proportions of bifidobacteria changed in the colonic fluid over time in this study, with an inverse relationship in *Clostridium* dominance. Bifidobacteria numbers were stable over the course of the study (>10^9^), indicating that the *Clostridium* population was unstable in the colon, possibly due to the influence of oxygen.

A recent study reported that the microbiota of the human adult ileum is relatively unstable and less complex when compared with the colonic microbiota (Booijink et al. 2010). Once again, the authors did not detect bifidobacteria in the adults, aged 41–74 and suffering from either ulcerative colitis or Crohn's disease. The authors also highlighted the number of novel phylotypes in the ileal microbiota which were unclassified, a phenomenon observed in this study in the colonic fluid. Interestingly, they highlighted the high relative abundance of *Streptococcus* and *Veillonella* in the ileal fluid of ulcerative colitis or Crohn's disease patients (Booijink et al. 2010), while we noted in this study an increase in the relative abundance of *Streptococcus* and *Veillonella* in the ileostomy infant. We did record bifidobacterial dominance in both the ileum and colon; however, the ileum was also dominated by *Streptococcus* and Enterobacteriaceae in both ileal and subsequent fecal samples, while the colonic fluid was dominated by bifidobacteria and clostridia, in addition to a large number of sequences which were not classified at genus level.

In conclusion, we found that the human infant ileum and colon are dominated by bifidobacteria and the microbiota of the neonatal ileum/colon in ileostomy/colostomy infants is dynamic and unstable, with large changes observed at genus level over the duration of this study. The absence of the strict anaerobes, Bacteroides, and the instability of *Clostridium* suggest that the gut is exposed to oxygen following ileostomy/colostomy which modifies microbiota composition.
